# Accurate non‐invasive diagnosis and staging of non‐alcoholic fatty liver disease using the urinary steroid metabolome

**DOI:** 10.1111/apt.15710

**Published:** 2020-04-16

**Authors:** Ahmad Moolla, Jasper de Boer, David Pavlov, Amin Amin, Angela Taylor, Lorna Gilligan, Beverly Hughes, John Ryan, Eleanor Barnes, Zaki Hassan‐Smith, Jane Grove, Guruprasad P. Aithal, An Verrijken, Sven Francque, Luc Van Gaal, Matthew J. Armstrong, Phillip N. Newsome, Jeremy F. Cobbold, Wiebke Arlt, Michael Biehl, Jeremy W. Tomlinson

**Affiliations:** ^1^ Oxford UK; ^2^ Groningen The Netherlands; ^3^ Birmingham UK; ^4^ Nottingham UK; ^5^ Antwerp Belgium; ^6^ Birmingham UK

## Abstract

**Background:**

The development of accurate, non‐invasive markers to diagnose and stage non‐alcoholic fatty liver disease (NAFLD) is critical to reduce the need for an invasive liver biopsy and to identify patients who are at the highest risk of hepatic and cardio‐metabolic complications. Disruption of steroid hormone metabolic pathways has been described in patients with NAFLD.

**Aim(s):**

To assess the hypothesis that assessment of the urinary steroid metabolome may provide a novel, non‐invasive biomarker strategy to stage NAFLD.

**Methods:**

We analysed the urinary steroid metabolome in 275 subjects (121 with biopsy‐proven NAFLD, 48 with alcohol‐related cirrhosis and 106 controls), using gas chromatography‐mass spectrometry (GC‐MS) coupled with machine learning‐based Generalised Matrix Learning Vector Quantisation (GMLVQ) analysis.

**Results:**

Generalised Matrix Learning Vector Quantisation analysis achieved excellent separation of early (F0‐F2) from advanced (F3‐F4) fibrosis (AUC receiver operating characteristics [ROC]: 0.92 [0.91‐0.94]). Furthermore, there was near perfect separation of controls from patients with advanced fibrotic NAFLD (AUC ROC = 0.99 [0.98‐0.99]) and from those with NAFLD cirrhosis (AUC ROC = 1.0 [1.0‐1.0]). This approach was also able to distinguish patients with NAFLD cirrhosis from those with alcohol‐related cirrhosis (AUC ROC = 0.83 [0.81‐0.85]).

**Conclusions:**

Unbiased GMLVQ analysis of the urinary steroid metabolome offers excellent potential as a non‐invasive biomarker approach to stage NAFLD fibrosis as well as to screen for NAFLD. A highly sensitive and specific urinary biomarker is likely to have clinical utility both in secondary care and in the broader general population within primary care and could significantly decrease the need for liver biopsy.

## 1 INTRODUCTION

Ectopic fat deposition in the liver, known as non‐alcoholic fatty liver disease (NAFLD), affects up to 30% of the worldwide population, up to 70% of patients with type 2 diabetes mellitus (T2D) and more than 90% of patients undergoing weight loss surgery.^1^ By 2025, it is estimated that NAFLD will be the leading cause of liver failure and leading indication for liver transplantation worldwide.^2^, ^3^ Despite the impact upon the liver, most morbidity and mortality in patients with NAFLD is driven through adverse cardiovascular outcomes.^4^ There is now clear evidence that morbidity and mortality (both cardiovascular and liver) increase with progressive disease that is driven by the degree of inflammation and fibrosis as well as development of T2D and continued weight gain.^4^, ^5^

Non‐alcoholic fatty liver disease is often asymptomatic until its late stages when either hepatic decompensation or cardiovascular complications may become apparent. Accurate and early staging is therefore important to determine the risk of complications and to guide the most appropriate management strategy. The current reference standard for staging liver fibrosis in patients with NAFLD remains liver biopsy, which is invasive, associated with morbidity, resource intensive and samples only a very small fraction of the liver and thus may be prone to sampling error.

Routine interpretation of liver biochemistry is unhelpful in staging NAFLD; 50% of patients with advanced fibrosis or cirrhosis have entirely normal liver chemistry.^6^ Faced with this challenge, several non‐invasive tools, including serological, clinical and imaging‐based markers and algorithms, have been developed attempting to reduce the need for liver biopsy to stage NAFLD.^7^ However, to date, none of these approaches have been shown to be sufficiently robust to replace liver biopsy. Most have good negative predictive value, although their sensitivity and positive predictive value are relatively poor.

Steroid hormones are primarily synthesised in the adrenal glands and gonads, however, the majority of their metabolism (primarily to inactive metabolites) occurs within the liver with subsequent excretion in the urine. Based on the paradigm of glucocorticoid excess (Cushing's syndrome) in which patients develop a florid metabolic phenotype characterised by obesity, insulin resistance, T2D and NAFLD,^8^ specific steroid metabolic pathways have been shown to be dysregulated in patients with NAFLD. These include the activities of the enzymes 11β‐hydroxysteroid dehydrogenase type 1 (11β‐HSD1), which regenerates cortisol (F) from inactive cortisone (E), and the A‐ring reductases (5α‐ and 5β‐reductase, 5αR/5βR), which inactivate cortisol to tetrahydrocortisol metabolites (5αTHF and THF).^9^, ^10^ However, these studies have been small and did not examine their relationship with histological disease stage. In addition, the complexities of hepatic steroid hormone metabolism means that specific ratios are influenced by the activities of multiple enzymes rendering their interpretation challenging.

We therefore proposed to test the hypothesis that the urinary steroid metabolome provides an accurate and dynamic reflection of steroid hormone metabolism within the liver, and that this in turn will be influenced by NAFLD disease stage. Through the adoption of interpretable machine learning algorithms, which simultaneously permitted specific pathway interrogation as well as a global analysis, we aimed to investigate whether the urinary steroid metabolome offered the potential to accurately and non‐invasively diagnose and stage NAFLD.

## 2 PATIENTS AND METHODS

Clinical data and urine samples were collected from 275 subjects; 169 patients with established liver disease (NAFLD n = 121; alcohol n = 48) and 106 controls without known liver disease. All samples were collected with full informed consent and national research ethical committee approval (refs. 09/H0403/1, 12/WM/0288, 07/H1211/168, 09/H604/20, and 10/H0402/23). All patients with NAFLD had liver biopsy staging performed, except in six patients where a diagnosis of cirrhosis was made using established clinical criteria (clinical examination, platelets and liver function blood tests, liver imaging and hepatic elastography). Determination of control status was established by review of medical history and the absence of risk factors for any known liver disease. Control subjects with abnormal liver chemistry or with elevated tests for liver disease severity (see below) were excluded from the analysis. Scores for non‐invasive markers of liver fibrosis were defined and calculated as follows:

- AST‐to‐Platelet Ratio Index = AST (IU/L)/(upper limit of normal)/platelet count (×10^9^/L) × 100
- Fibrosis‐4 score (FIB‐4) = age × AST (IU/L)/platelet count (×10^9^/L) × √ALT (IU/L)
- AST/ALT ratio = AST (IU/L)/ALT (IU/L)
- NAFLD fibrosis score = −1.675 + 0.037 × age (y) + 0.094 × body mass index (BMI) (kg/m^2^) + 1.13 × Impaired fasting glucose or T2D (yes = 1, no = 0) + 0.99 × AST/ALT ratio − 0.013 × platelet count (×10^9^/L) − 0.66 × albumin (g/dL)
- BARD score = sum (BMI >28 kg/m^2^ = 1, AST/ALT ratio >0.8 = 2, T2D = 1)

### 2.1 Histological liver staging of NAFLD

Liver biopsies were performed as part of clinical care in patients with NAFLD. NAFLD Activity Score (NAS) (including the individual components of lobular, inflammation, steatosis, hepatocyte ballooning and fibrosis) as well as NAFLD fibrosis stage (F0‐F4) were assessed by the Kleiner staging system.^11^ F0 represents the absence of fibrosis, F1 portal or peri‐sinusoidal fibrosis, F2 portal/peri‐portal and peri‐sinusoidal fibrosis, F3 septal or bridging fibrosis and F4 cirrhosis.

### 2.2 Urinary steroid metabolite analysis

Spot urine samples from a single void of urine were collected from each subject and stored at −80°C. Measurement of urinary steroid metabolites was undertaken using gas chromatography/mass spectrometry (GC/MS) as described previously.^12^

In brief, free and conjugated steroids were extracted from 1mL of urine via a 5‐step extraction method. Solid‐phase extraction of free and conjugated steroids was performed. Steroid conjugates underwent enzymatic hydrolysis followed by solid‐phase re‐extraction of steroids, chemical derivatisation to form ethers and finally liquid‐liquid extraction. GC/MS was undertaken on an Agilent 5973 MSD single‐quadrupole gas chromatography mass spectrometer (Agilent) instrument allowing quantification of up to 32 steroid metabolites, with representation of major steroids and their metabolites from all the adrenal‐derived steroid hormone classes (androgens, glucocorticoids and mineralocorticoids [Table S1]). Steroids were identified in SIM (single ion monitoring) mode and quantified relative to authentic reference standards. Multi‐steroid profiling includes the metabolites of all precursors and end products of the major steroid hormone classes (androgens, glucocorticoids and mineralocorticoids; Table S1.

For each urine sample, a creatinine correction was made (see below) in an attempt to adjust for differing times and durations of collection (urinary creatinine is excreted at a relatively constant rate and is widely used as a corrective factor). Data were expressed as μg steroid/g urinary creatinine. A separate analysis of uncorrected data expressed as μg steroid/1000 mL urine is presented in the supplementary data.

Measurement of individual steroid hormone concentrations and their metabolites permitted assessment of individual steroid metabolic pathways based on the analysis of ‘*precursor to product metabolite’* ratios. All individual steroid data were log transformed (Log10) prior to analysis. Product‐to‐substrate metabolite ratios investigating specific pathways of glucocorticoid metabolism were calculated as follows:

- 11β‐HSD1 activity = (THF + 5αTHF)/THE
- A‐ring reductase activity = 5αTHF/THF

In addition, we calculated total glucocorticoid excretion as the sum of the following steroid metabolites: Total Cortisol (F) Metabolites = 6β‐hydroxy‐cortisol + tetrahydrocortisol (THF) + 5α‐tetrahydrocortisol (5αTHF) + α‐cortol + β‐cortol + 11β‐hydroxyetiocholanolone + cortisone (E) + tetrahydrocortisone (THE) + α‐cortolone + β‐cortolone + 11‐oxoetiocholanolone.

### 2.3 Urinary creatinine assay

Urinary creatinine measurement was performed using the QuantiChromTM Creatinine Assay Kit (DICT‐500, Universal Biologicals). Five microlitre of either standard (50 mg/dL) or urine was mixed with 200 μL of working reagent in a 96‐well plate. Optical density was read at 0 minute and 5 minutes at an absorbance of 490 nm on a VersaMax Plate Reader (Molecular Devices) and the creatinine concentration (mg/dL) was calculated for each urine sample in duplicate as per the manufacturer guidance. A mean creatinine value (mg/dL) was calculated from a minimum of two independent assays.

### 2.4 Generalised Matrix learning vector quantisation computational analysis

Learning Vector Quantisation (LVQ) is a machine learning technique that extracts typical class representatives or prototypes from training data.^13^ For our application this translated to one typical steroid profile per disease stage. These prototypes can be used to classify a steroid profile with unknown disease stage: the most probable disease stage is determined by selecting the class of the prototype that is most similar to the new profile. The dis‐similarity of a given steroid profile and a prototype is defined by a distance measure, for example, the conventional Euclidean distance. In Generalised Matrix Learning Vector Quantisation (GMLVQ),^14^ however, the distance metric itself is adaptive and optimised together with the prototypes in the same data‐driven training process. This metric is defined through a matrix of adaptive parameters, termed the relevance matrix. Its diagonal elements quantify the importance of individual steroids in the classification scheme. Details of the GMLVQ analysis are presented in detail in the supplementary appendix, including the use of receiver operating charcteristic curves ^15^.

### 2.5 Statistical analysis

Steroid metabolite ratio data are graphically represented as mean and standard error of the mean using GraphPad Prism version 7.02 (GraphPad Software). Individual steroid data and steroid ratios were compared among control, early fibrosis (F0‐F2) and advanced fibrosis (F3‐F4) groups using the Kruskal‐Wallis nonparametric test and pair‐wise multiple comparisons between groups were undertaken using Dunn's post hoc test. Significance was determined as *P* < 0.05.

## 3 RESULTS

A total of 275 individuals were recruited into the study (106 controls, 121 with NAFLD and 48 with alcohol‐related cirrhosis). Demographic details as well as biochemical and histological assessment are presented in Table 1 and Table S3. There was no significant difference in gender ratios between groups, although age was significantly different between all three groups. Those with advanced fibrosis were older than the other two groups, although controls were older than those with early fibrosis (F0‐F2). All groups had a mean BMI in the obese range (BMI >30 kg/m^2^); BMI was highest in those with early fibrosis.

**TABLE 1:** Demographic details of 227 subjects: 106 control and 121 individuals with biopsy‐proven NAFLD stratified by fibrosis stage (F0‐2 vs F3‐4)

	Control	F0‐2	F3‐4	*P*‐value
N (m/f) (males, %)	106 (41/65) (38.7)	39 (20/19) (51.3)	82 (39/43) (47.6)	0.29
Age, y	55.5 ± 11.1	45.6 ± 12.0*	61.8 ± 10.8*^,^**	<0.0001
BMI, kg/m^2^	30.7 ± 5.8	38.5 ± 7.0*	33.7 ± 5.8*^,^**	<0.0001
Proportion with type 2 diabetes, %	3.8	30.8*	63.4*^,^**	<0.0001
HbA1c, mmol/mol	38.6 ± 10.4	40.8 ± 8.2	50.0 ± 13.5*^,^**	<0.0001
Platelets, 10 y^2^/L	n/a	242.5 ± 64.2	183.9 ± 67.0**	<0.0001
ALT, IU/L	13.2 ± 8.7	63.4 ± 51.4*	49.9 ± 36.9*	<0.0001
AST, IU/L	n/a	34.4 ± 22.0	49.1 ± 31.8**	0.0006
Fib‐4 score	n/a	0.931 ± 0.7	2.61 ± 1.7**	<0.0001
NAFLD fibrosis score	n/a	1.9 ± 1.2	3.8 ± 1.6**	<0.0001
NAS score (0‐8)	n/a	4.0 ± 1.7	4.7 ± 1.3**	0.029
Proportion with NAS score ≥5, %	n/a	42.1	63.1	0.07

Note

Data expressed are mean ± standard deviation (unless otherwise stated). Where applicable, *P*‐value stated in the final column is the summary ANOVA value when all three groups are compared (*P < 0.05 vs control; **P < 0.05 vs F0‐2).

Abbreviations: BMI, body mass index; FIB‐4, fibrosis‐4 score; NAFLD, non‐alcoholic fatty liver disease; NAS, NAFLD Activity Score.

### 3.1 Increased 11β‐hydroxysteroid dehydrogenase type 1 and 5α‐reductase activity in patients with NAFLD and advanced fibrosis

**FIGURE 1:**
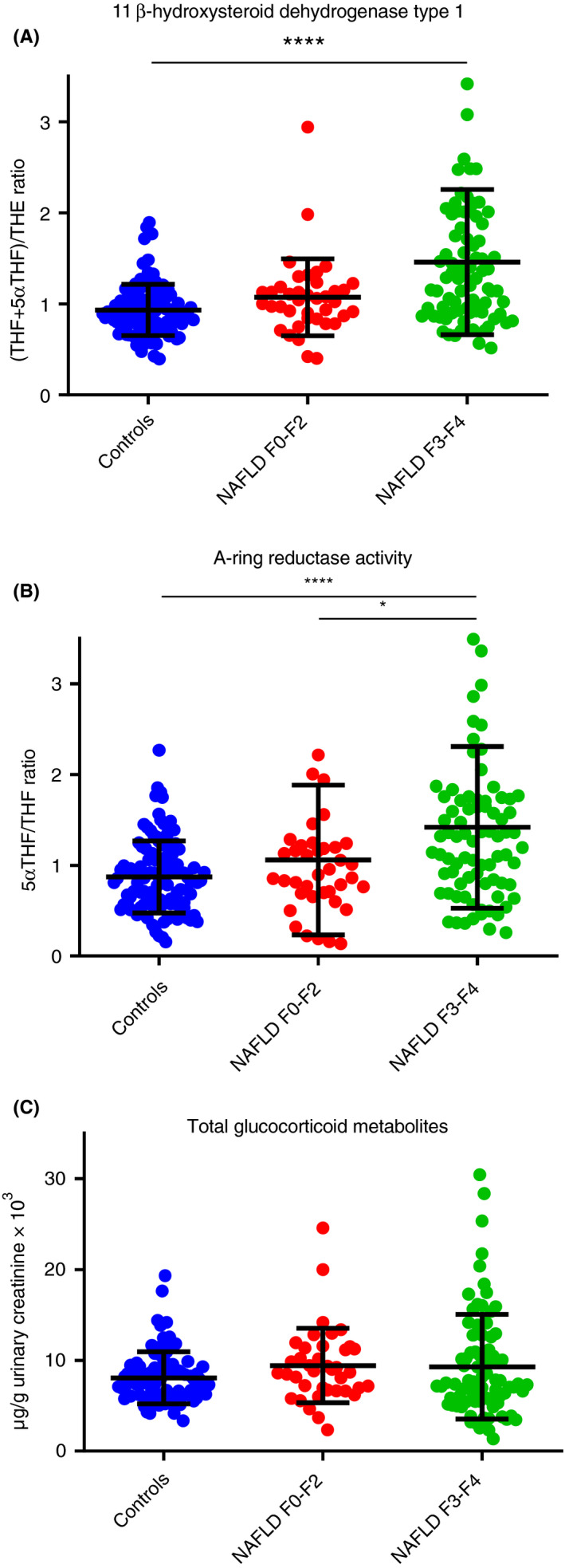
Total Glucocorticoid Metabolites, 11β‐hydroxysteroid dehydrogenase type 1 and 5α‐reductase activities based on urinary multi‐steroid profiling by GC‐MS. Statistical analysis performed on log‐transformed steroid values or ratios. Data shown: mean ± SD. Two and 4 data points not shown in (A) and (B), respectively, for graphical purposes. Both 11β‐hydroxysteroid dehydrogenase type 1 (A) and 5α‐reductase (B) activities are increased in patients with NAFLD with advanced fibrosis, although not in those with mild disease when compared with controls. Total glucocorticoid metabolite production was not different across the spectrum of NAFLD or in comparison with controls (****P < 0.0001, *P < 0.05). NAFLD, non‐alcoholic fatty liver disease

Data for specific steroid metabolites and ratios indicative of specific enzyme activity are presented in Table S2. In our cohort, 11β‐HSD1 activity, as reflected by the (THF + 5αTHF)/THE ratio, was increased, consistent with enhanced cortisol regeneration from inactive cortisone, in patients with NAFLD and advanced fibrosis (F3‐4) (Figure 1A), although not in those with early fibrosis (F0‐2). In parallel, we observed an increase in systemic 5α‐reductase activity, which enhances cortisol clearance (Figure 1B). There was no change in total glucocorticoid metabolite production (Figure 1C).

### 3.2 GMLVQ analysis of the urinary steroid metabolome can distinguish early from advanced fibrosis

Analysis of data using individual steroid metabolites and ratios demonstrated significant overlap across all fibrosis groups and therefore there was limited potential to be able to correctly determine NAFLD disease stage. We therefore adopted a global approach using GMLVQ to analyse all 32 urinary steroids and metabolites (Figure S1A) based on the generation of prototype steroid profiles (Figure S1B) and a relevance matrix which indicates the importance of individual steroids to the GMLVQ classifier (Figure S1C).

**FIGURE 2:**
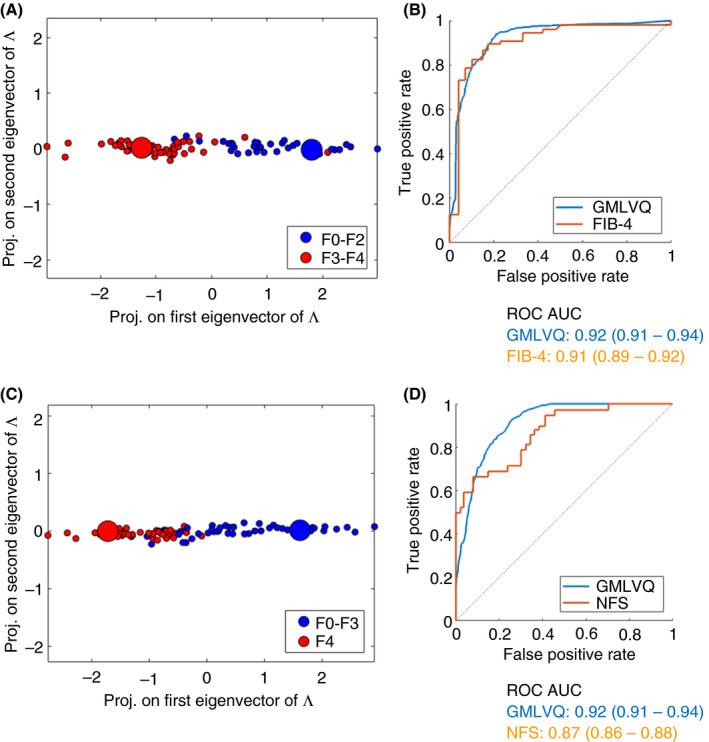
GMLVQ* analysis, including steroid values, BMI and age, permits very good separation between early and advanced fibrosis (F0‐2 vs F3‐4) in patients with NAFLD (A). ROC AUC analysis is presented in comparison with FIB‐4 (the best‐performing serological test in this analysis) (B). The performance of GMLVQ* to identify patients with cirrhosis (F0‐3 vs F4) is also very good (C), with ROC AUC analysis demonstrating significant improvement in diagnostic ability when compared with NAFLD fibrosis score (the best‐performing serological test in this analysis) (D). BMI, body mass index; FIB‐4, fibrosis‐4 score; GMLVQ, Generalised Matrix Learning Vector Quantisation; NAFLD, non‐alcoholic fatty liver disease; ROC, receiver operating characteristics

Generalised Matrix Learning Vector Quantisation performance was further enhanced by the inclusion of both age and BMI into the model (GMLVQ*) (Table 2). To address the binary problem of identifying those individuals with established NAFLD who have either early (F0‐2) vs advanced (F3‐4) fibrosis, 2D representative plots were produced as shown in Figure 2A demonstrating good separation. Corresponding area under the curve (AUC) analysis of the receiver operating characteristics (ROC) curves suggested that urinary steroid GMLVQ and GMLVQ* analysis performed as well as the established non‐invasive serum marker algorithm, Fib‐4 (Figure 2B) (Table 2).

**TABLE 2:** Comparison of GMLVQ analysis of urinary steroid metabolites vs serum assessments using Fib4 and NAFLD fibrosis scores (analysis of samples corrected for urinary creatinine)

Clinical comparison (NAFLD stage)	AUC ROC (95% confidence intervals)					
	NAFLD Fibrosis score	FIB‐4	GMLVQ (32 steroids)	GMLVQ* (32 steroids, age, BMI)	GMLVQ‐10 (top 10 steroid metabolites)	GMLVQ‐10* (top 10 steroid metabolites, age, BMI)
*F0‐F2* vs *F3‐F4*	0.87 (0.86‐0.88)	0.91 (0.89‐0.92)	0.89 (0.87‐0.90)	0.92 (0.91‐0.94)	0.87 (0.85‐0.88)	0.92 (0.91‐0.94)
*F0‐F3* vs *F4*	0.87 (0.86‐0.88)	0.84 (0.83‐0.85)	0.87 (0.85‐0.89)	0.92 (0.91‐0.94)	0.85 (0.83‐0.87)	0.90 (0.89‐0.92)
*Controls* vs *F0‐F4*			0.93 (0.92‐0.94)	0.94 (0.92‐0.95)	0.92 (0.91‐0.93)	0.94 (0.93‐0.96)
*Controls* vs *F3‐F4*			0.99 (0.98‐0.99)	0.98 (0.97‐0.98)	0.99 (0.98‐0.99)	0.98 (0.98‐0.99)
*Controls* vs *F4*			1.00 (1.00‐1.00)	1.00 (1.00‐1.00)	1.00 (1.00‐1.00)	1.00 (0.99‐1.00)

Abbreviations: BMI, body mass index; FIB‐4, fibrosis‐4 score; GMLVQ, Generalised Matrix Learning Vector Quantisation; NAFLD, non‐alcoholic fatty liver disease; ROC, receiver operating characteristics.

Patients with liver cirrhosis are at a higher risk of developing hepatocellular carcinoma and hepatic decompensation and therefore require active monitoring and surveillance. GMLVQ and GMLVQ* were able to accurately identify those patients with NAFLD cirrhosis (F0‐3 vs F4) and out‐performed non‐invasive serological assessments including NAFLD fibrosis score and Fib‐4 (Figure 2C,D, Table 2).

### 3.3 GMLVQ analysis of the urinary steroid metabolome has excellent potential to identify patients with advanced NAFLD in the general population

**FIGURE 3:**
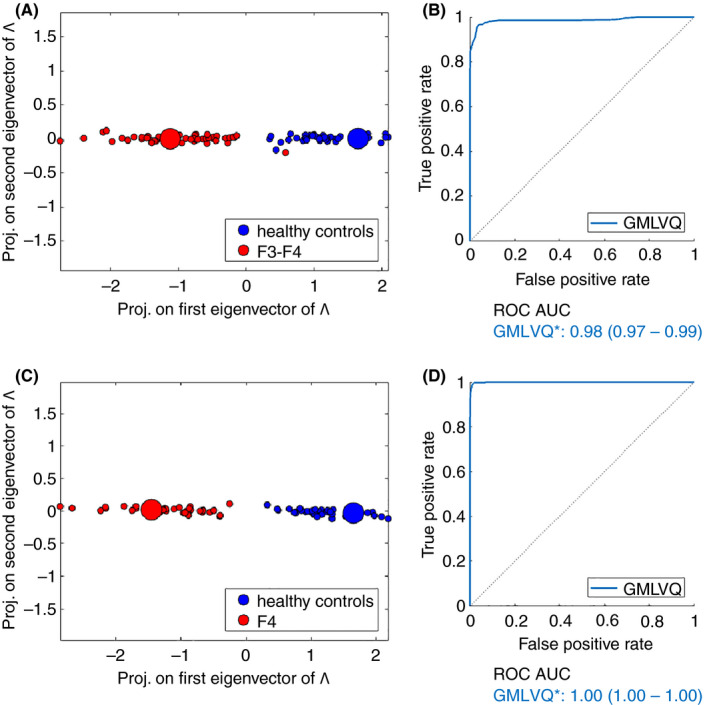
GMLVQ* analysis, including steroid values, BMI and age, has excellent potential utility as a screening tool to identify individuals with advanced NAFLD fibrosis within the general population. There was excellent separation between controls and those with advanced NAFLD fibrosis (A) with the corresponding ROC AUC analysis (B). The performance of GMLVQ* to identify patients with NAFLD cirrhosis in the general population (control vs F4) is excellent with perfect separation (C and D). BMI, body mass index; GMLVQ, Generalised Matrix Learning Vector Quantisation; NAFLD, non‐alcoholic fatty liver disease; ROC, receiver operating characteristics

Both GMLVQ and GMLVQ* demonstrated excellent separation and diagnostic ability in identifying patients with advanced NAFLD when compared with controls (Figure 3A,B). When used to identify those patients with NASH cirrhosis, there was perfect separation and AUC ROC = 1.0 (Figure 3C,D) (Table 2).

To determine if GMLVQ* of urinary steroid metabolite data could identify the underlying aetiology of cirrhosis, a further analysis comparing samples from patients with NAFLD cirrhosis to those from patients with alcohol‐related cirrhosis was performed (Table S3). GMLVQ* demonstrated good separation and diagnostic ability to differentiate the underlying aetiology of cirrhosis (AUC ROC = 0.83 [0.81‐0.85, 95% confidence intervals], Figure S2).

Additional analyses were also performed separating data by gender as well as comparing urinary steroid metabolites uncorrected for urinary creatinine. There was no impact of gender on the GMLVQ model performance when analysis was undertaken directly comparing men vs women. In addition, when gender was added as a variable into the GMLVQ analysis, there was no alteration in the performance of the model to predict stage of disease (data not shown). AUC ROC analysis was similar using data from samples where uncorrected steroid metabolite levels were expressed as μg steroid/1000 mL urine (Table S4). Furthermore, as NASH is an important feature in the disease spectrum of NAFLD, GMLVQ analysis was tested for its ability to predict NASH. GMLVQ analysis was unable to distinguishing between NASH (NAS >4) and non‐NASH in those with established NAFLD (Figure S3).

### 3.4 GMVLQ can be refined to include only 10 urinary steroid metabolites without significant loss in diagnostic performance

**FIGURE 4:**
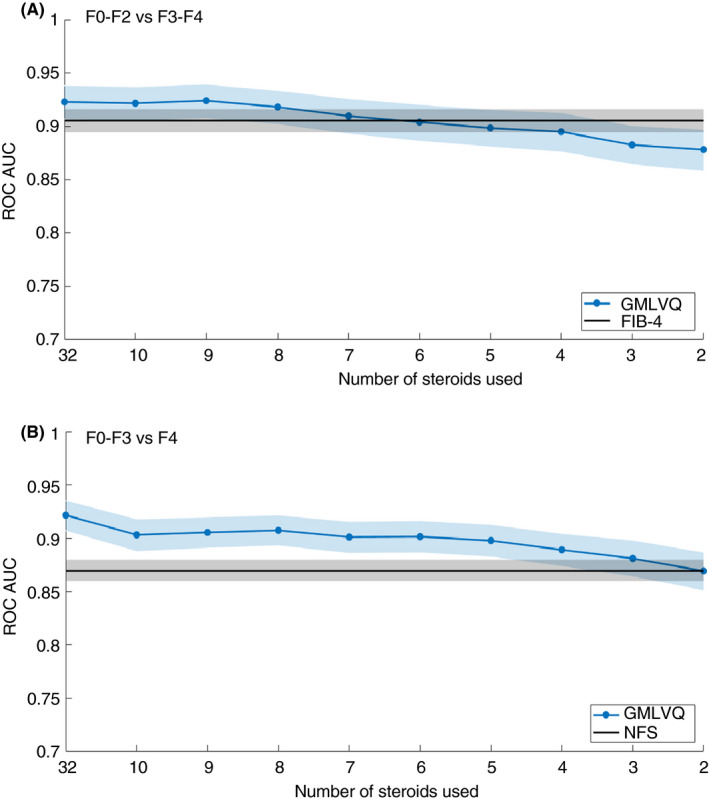
The ability of GMLVQ and GMLVQ* to identify advanced NAFLD fibrosis (F3‐4) (A) and cirrhosis (F4) (B) can be refined to a panel of approximately 10 specific steroid metabolites (GMLVQ‐10*) without significant reduction in diagnostic performance. GMLVQ, Generalised Matrix Learning Vector Quantisation; NAFLD, non‐alcoholic fatty liver disease

A further GMLVQ analysis was performed with sequential removal of the least discriminatory steroid metabolites. GMLVQ analysis was then compared against the best‐performing non‐invasive serum markers (Fib‐4 for F0‐2 vs F3‐4 and NAFLD fibrosis score for F0‐3 vs F4). Refining the model from 32 metabolites to 10 (GMLVQ‐10) did not result in any loss of diagnostic performance and GMLVQ analysis incorporating age and BMI using 10 steroid metabolites (GMLVQ‐10*) still out‐performed FIB‐4 (F0‐2 vs F3‐4) and NAFLD fibrosis score (F0‐3 vs F4) (Figure 4A,B, Table 3). In addition, the analysis of 10 most discriminatory steroids was still able to distinguish NAFLD cirrhosis from alcohol‐related cirrhosis (GMLVQ‐10*; AUC ROC = 0.82 [0.81‐0.84, 95% confidence intervals]). The 10 most discriminatory steroids that had the most impact in distinguishing each of the clinical comparisons (F0‐2 vs F3‐4; F0‐3 vs F4; Control vs F3‐4; Control vs F4) are shown in Table 3.

**TABLE 3:** GMLVQ analysis identifies the 10 most discriminatory steroid metabolites for distinguishing clinically relevant stages of NAFLD

Discriminatory ranking	NAFLD stage comparison			
	F0‐2 vs F3‐4	F0‐3 vs F4	Control vs F3‐4	Control vs F4
1	Etiocholanolone	Etiocholanolone	5α‐tetrahydro‐11‐dehydrocorticosterone	5α‐tetrahydro‐11‐dehydrocorticosterone
2	Dehydroepiandrosterone	Tetrahydrocorticosterone	11‐oxoetiocholanolone	11‐oxoetiocholanolone
3	5α‐tetrahydro‐11‐dehydrocorticosterone	5α‐tetrahydro‐11‐dehydrocorticosterone	Etiocholanolone	Etiocholanolone
4	Androstendione	Tetrahydro‐11 deoxycorticosterone	Cortisone	Cortisone
5	5α‐tetrahydrocorticosterone	Dehydroepiandrosterone	Pregnenediol	Tetrahydro‐11 deoxycorticosterone
6	Pregnenetriol	Androsterone	Pregnanetriol	Pregnenediol
7	Tetrahydro‐11 deoxycorticosterone	Tetrahydrocortisone	Tetrahydro‐11 deoxycorticosterone	Pregnanetriol
8	Tetrahydroaldosterone	Tetrahydrocortisol	11β‐hydroxyetiocholanolone	Tetrahydrocorticosterone
9	Cortisone	Pregnenetriol	Pregnanediol	Pregnanediol
10	11‐oxoetiocholanolone	5α‐tetrahydrocorticosterone	5α‐tetrahydrocorticosterone	5α‐tetrahydrocorticosterone

Abbreviations: GMLVQ, Generalised Matrix Learning Vector Quantisation; NAFLD, non‐alcoholic fatty liver disease.

## 4 CONCLUSIONS

We have demonstrated for the first time that urinary steroid metabolome profiling in spot urine samples combined with machine learning‐based analysis can accurately identify patients with NAFLD who have the most advanced stages of liver disease including cirrhosis (F3‐F4). This novel urinary biomarker algorithm performed better than, or equivalently to the FIB‐4 and NAFLD fibrosis scores, two commonly used noninvasive biomarkers in the evaluation of advanced liver fibrosis in patients with NAFLD.

The relationship between NAFLD fibrosis stage and clinical outcome is now well‐established.^4^ Furthermore, population‐based studies have suggested a high prevalence of undiagnosed advanced NAFLD in the general population,^16^, ^17^ and while screening is not currently advocated, identification of advanced fibrosis and cirrhosis would significantly alter patient management. If appropriate management strategies are to be implemented, investigative tools that do not carry the associated risks and limitations of liver biopsy are needed. There is therefore a pressing need for the development of accurate non‐invasive markers to determine stage of liver disease, fuelled, at least in part, by the poor performance of simple routine liver biochemistry.^6^

The number of potential tests that can be used to assess the risk of advanced fibrosis is large. Data from more than 20 different tests, algorithms or imaging platforms have been published^7^ and the large number of tests perhaps reflects the need for improved performance. The range of ROC AUC values is broad for many of these tests that are currently used in clinical practice, and although these can sometimes exceed 0.90, the majority of studies suggest values between 0.8 and 0.9. The potential use of a urinary test is novel, completely non‐invasive, easily accessible and acceptable to patients and may easily be performed across both primary and secondary care opening the possibility of more widespread use in screening the general population. Urine sampling has a higher degree of patient acceptability than venepuncture; it requires no specialist sampling equipment or personnel and with further development, rapid high‐throughput platforms may make urinary steroid GMLVQ analysis a cost‐effective approach.

Untargeted urinary metabolic profiling in small numbers of patients has been explored,^18^, ^19^ but its diagnostic ability has not been interrogated in detail. Volatile organic compound generated via the gut microbiome and excreted in the urine have been examined in a very small exploratory pilot study with AUC ROC values of 0.73 (0.45‐0.92) to distinguish advanced disease.^20^

The liver is the main site of steroid hormone metabolism and we have hypothesised that the assessment of urinary steroid metabolites may provide a functional assessment of liver that may differ according to NAFLD stage. Previous work has focussed on specific steroid pathways that appear to be dysregulated. For example, there is evidence for increased 11β‐HSD1 activity (as observed in our cohort) in patients with NASH, and it has been postulated that the resultant increased cortisol generation may serve as auto‐regulatory mechanism to limit local hepatic inflammation.^9^ In the same study (and contrasting with our data), 5α‐reductase activity (to clear metabolically cortisol through the generation of inactive tetrahydrocortisol) was decreased. The reasons for this discrepancy are not clear, although the small number of participants and the lack of detailed histological staging (including fibrosis stage) in the published studies need to be considered. An additional study has suggested liver fat content correlates with 5β‐reductase activity, catalysed by the enzyme AKRD1 that is almost exclusively expressed in the liver.^10^

There is clear biological plausibility in our approach. Manipulation of steroid metabolising enzyme activity can impact upon hepatic phenotype. Inhibition of 11β‐HSD1 decreases hepatic steatosis (albeit with a modest effect size).^21^ Similarly, combined 5α‐reductase type 1 and 2 inhibition with dutasteride increased hepatic triglyceride accumulation as well as driving insulin resistance.^22^, ^23^ Furthermore, inflammatory stimuli have been shown to regulate the expression and activity of steroid metabolising enzymes.^24^ Although precise role in steroid hormone metabolism is yet to be determined, the recent identification of specific genetic variants in 17β‐hydroxysteroid dehydrogenase type 13 (HSD17B13) adds further plausibility to our approach as this enzyme appears to protect from the development of chronic liver disease and hepatocellular carcinoma with several studies demonstrating increased expression in NAFLD add further plausibility to our approach.^25^, ^26^ In addition, the ability of ethanol to regulate steroid hormone metabolising enzymes has been described^27^ and this may underpin the ability of our analysis to distinguish NAFLD from alcohol‐related cirrhosis.

Urinary steroid metabolome analysis using GMLVQ has been used to help differentiate benign from malignant adrenal tumours,^28^ but its use in the context of NAFLD is entirely novel. Data from our study (AUC ROC >0.9) would suggest excellent potential for GMLVQ analysis of urinary steroids as a strategy for accurate identification of those patients with advanced fibrosis. This may be relevant for identifying patients within the general population that have the most advanced liver disease who are at high risk of cardiovascular and hepatic co‐morbidities and complications. Estimates suggest that prevalence of compensated cirrhosis is likely to rise in the general population by more than 150% in some countries over the next 10‐15 years and therefore identification of these patients is of huge clinical significance.^29^

Our study is not without limitations. The data that we have presented are from a modest‐sized cohort of patients, although biomarker exploration in the context of NAFLD has typically been undertaken in cohorts of this size.^30^ This was a retrospective study and the sizes and clinical characteristics did differ between groups; age, BMI and the prevalence of T2D were different. These are all important variables that need to be considered in determining the risk of advanced NAFLD. Age and BMI were included in the refined GMLVQ* model, however, the addition of variables relating to the presence or absence of T2D or the glycated haemoglobin numerical data did not improve the performance of the model (data not shown). The NAFLD cohort in this study had a higher prevalence of advanced fibrotic disease than would be expected in the general population, probably reflecting the fact that most patients were identified in secondary care. Furthermore, this may explain why some of the comparator non‐invasive tests, such as the Fib‐4, performed better than has been observed in the published literature. Finally, the current methodology of GC‐MS is time consuming and labour intensive. However, it is entirely plausible that our analysis can be transferred to a high‐throughput, more cost‐ and time‐efficient liquid chromatography tandem mass spectrometry platform. With these limitations in mind, there is a clear need to validate the findings from this study in a larger independent cohort with detailed histological staging of disease and comprehensive clinical characterisation.

In conclusion, we have presented proof of principle for an entirely novel approach to stage NAFLD. Adopting machine learning algorithms has allowed the generation of a meaningful biomarker that may have excellent future clinical utility in the assessment and staging of NAFLD, both in secondary care and also in the broader general population and may reduce the need for liver biopsy. A prospective test validation study is now required prior to roll out of this novel, non‐invasive approach into clinical practice.

## AUTHORSHIP

*Guarantor or the article*: JWT.

*Author contributions*: JWT, AM, WA and MB designed the study, collated and analysed the data. JDeB, DP and MB performed the GMLVQ analysis; AT, LG and BH analysed the clinical samples. AM, JR, EB, ZH‐S, JG, GPA, AV, SF, LVG, MJA, PNN and JFC all provided clinical samples. JWT and AM drafted the manuscript. All authors contributed to the editing of the manuscript and all have approved the final version.

## Supporting information

Table S1‐S4‐Fig S1‐S3

## Appendix A

### The complete list of authors and affiliations

Ahmad Moolla, Jeremy W. Tomlinson: Oxford Centre for Diabetes Endocrinology & Metabolism (OCDEM), NIHR Oxford Biomedical Research Centre, University of Oxford, Churchill Hospital, Oxford, UK; Jasper de Boer, David Pavlov, Michael Biehl: Bernoulli Institute for Mathematics, Computer Science and Artificial Intelligence, University of Groningen, Groningen, The Netherlands; Amin Amin, Angela Taylor, Lorna Gilligan, Beverly Hughes, Zaki Hassan‐Smith, Wiebke Arlt: Institute of Metabolism and Systems Research, University of Birmingham, Birmingham, UK; John Ryan, Eleanor Barnes, Jeremy F. Cobbold: Translational Gastroenterology Unit, University of Oxford, Oxford, UK; Jane Grove, Guruprasad P Aithal: Nottingham Digestive Diseases Centre, Queen’s Medical Centre, University of Nottingham, Nottingham, UK; Guruprasad P. Aithal: NIHR Nottingham Biomedical Research Centre, Nottingham University Hospitals NHS Trust and the University Of Nottingham, Nottingham, UK; An Verrijken, Luc Van Gaal: Department of Endocrinology, Diabetology and Metabolism, Antwerp University Hospital, Antwerp, Belgium; An Verrijken, Sven Francque, Luc Van Gaal: Laboratory of Experimental Medicine and Paediatrics, Faculty of Medicine and Health Sciences, University of Antwerp, Antwerp, Belgium; Sven Francque: Department of Gastroenterology and Hepatology, Antwerp University Hospital, Antwerp, Belgium; Matthew J. Armstrong, Phillip N. Newsome: National Institute for Health Research Birmingham Biomedical Research Centre, University Hospitals Birmingham NHS Foundation Trust and University of Birmingham, Birmingham, UK; Centre for Liver and Gastrointestinal Research, Institute of Immunology and Immunotherapy, University of Birmingham, UK; Liver Unit, University Hospitals Birmingham NHS Foundation Trust, Birmingham, UK

## References

[1] Williamson RM , Price JF , Glancy S , et al. Prevalence of and risk factors for hepatic steatosis and nonalcoholic Fatty liver disease in people with type 2 diabetes: the Edinburgh Type 2 Diabetes Study. Diabetes Care. 2011;34:1139‐1144. 21478462 10.2337/dc10-2229 PMC3114489

[2] Wong RJ , Aguilar M , Cheung R , et al. Nonalcoholic steatohepatitis is the second leading etiology of liver disease among adults awaiting liver transplantation in the United States. Gastroenterology. 2015;148:547‐555. 25461851 10.1053/j.gastro.2014.11.039

[3] Haldar D , Kern B , Hodson J , et al. Outcomes of liver transplantation for non‐alcoholic steatohepatitis: a European Liver Transplant Registry study. J Hepatol. 2019;71:313‐322. 31071367 10.1016/j.jhep.2019.04.011 PMC6656693

[4] Dulai PS , Singh S , Patel J , et al. Increased risk of mortality by fibrosis stage in nonalcoholic fatty liver disease: systematic review and meta‐analysis. Hepatology. 2017;65:1557‐1565. 28130788 10.1002/hep.29085 PMC5397356

[5] McPherson S , Hardy T , Henderson E , Burt AD , Day CP , Anstee QM . Evidence of NAFLD progression from steatosis to fibrosing‐steatohepatitis using paired biopsies: implications for prognosis and clinical management. J Hepatol. 2015;62:1148‐1155. 25477264 10.1016/j.jhep.2014.11.034

[6] Verma S , Jensen D , Hart J , Mohanty SR . Predictive value of ALT levels for non‐alcoholic steatohepatitis (NASH) and advanced fibrosis in non‐alcoholic fatty liver disease (NAFLD). Liver Int. 2013;33:1398‐1405. 23763360 10.1111/liv.12226

[7] European Association for the Study of the Liver (EASL); European Association for the Study of Diabetes (EASD); European Association for the Study of Obesity (EASO) . EASL‐EASD‐EASO Clinical Practice Guidelines for the management of non‐alcoholic fatty liver disease. Diabetologia. 2016;59:1121‐1140. 27053230 10.1007/s00125-016-3902-y

[8] Rockall AG , Sohaib SA , Evans D , et al. Hepatic steatosis in Cushing's syndrome: a radiological assessment using computed tomography. Eur J Endocrinol. 2003;149:543‐548. 14640995 10.1530/eje.0.1490543

[9] Ahmed A , Rabbitt E , Brady T , et al. A switch in hepatic cortisol metabolism across the spectrum of non alcoholic fatty liver disease. PLoS One. 2012;7:e29531. 22363403 10.1371/journal.pone.0029531 PMC3282715

[10] Westerbacka J , Yki‐Jarvinen H , Vehkavaara S , et al. Body fat distribution and cortisol metabolism in healthy men: enhanced 5beta‐reductase and lower cortisol/cortisone metabolite ratios in men with fatty liver. J Clin Endocrinol Metab. 2003;88:4924‐4931. 14557475 10.1210/jc.2003-030596

[11] Kleiner DE , Brunt EM , Van Natta M , et al. Design and validation of a histological scoring system for nonalcoholic fatty liver disease. Hepatology. 2005;41:1313‐1321. 15915461 10.1002/hep.20701

[12] Shackleton CHL . Mass spectrometry in the diagnosis of steroid‐related disorders and hypertension research. J Steroid Biochem Mol Biol. 1993;45:127‐140. 8481337 10.1016/0960-0760(93)90132-g

[13] Biehl M , Hammer B , Villmann T . Prototype‐based models in machine learning. Wiley Interdiscip Rev Cognit Sci. 2016;7:92‐111. 26800334 10.1002/wcs.1378

[14] Schneider P , Biehl M , Hammer B . Adaptive relevance matrices in learning vector quantization. Neural Comput. 2009;21:3532‐3561. 19764875 10.1162/neco.2009.11-08-908

[15] Fawcett T . An introduction to ROC analysis. Pattern Recogn Lett. 2006;27:861‐874.

[16] Caballeria L , Pera G , Arteaga I , et al. High prevalence of liver fibrosis among european adults with unknown liver disease: a population‐based study. Clin Gastroenterol Hepatol. 2018;16:1138‐1145.e5. 29452268 10.1016/j.cgh.2017.12.048

[17] Harman DJ , Ryder SD , James MW , et al. Obesity and type 2 diabetes are important risk factors underlying previously undiagnosed cirrhosis in general practice: a cross‐sectional study using transient elastography. Aliment Pharmacol Ther. 2018;47:504‐515. 29210096 10.1111/apt.14463

[18] Troisi J , Pierri L , Landolfi A , et al. Urinary metabolomics in pediatric obesity and NAFLD identifies metabolic pathways/metabolites related to dietary habits and gut‐liver axis perturbations. Nutrients. 2017;9:485. 10.3390/nu9050485 PMC5452215 28492501

[19] Dong S , Zhan ZY , Cao HY , et al. Urinary metabolomics analysis identifies key biomarkers of different stages of nonalcoholic fatty liver disease. World J Gastroenterol. 2017;23:2771‐2784. 28487615 10.3748/wjg.v23.i15.2771 PMC5403757

[20] Arasaradnam RP , McFarlane M , Daulton E , et al. Non‐invasive distinction of non‐alcoholic fatty liver disease using urinary volatile organic compound analysis: early results. J Gastrointestin Liver Dis. 2015;24:197‐201. 26114180 10.15403/jgld.2014.1121.242.ury

[21] Stefan N , Ramsauer M , Jordan P , et al. Inhibition of 11beta‐HSD1 with RO5093151 for non‐alcoholic fatty liver disease: a multicentre, randomised, double‐blind, placebo‐controlled trial. Lancet Diabetes Endocrinol. 2014;2:406‐416. 24795254 10.1016/S2213-8587(13)70170-0

[22] Hazlehurst JM , Oprescu AI , Nikolaou N , et al. Dual‐5alpha‐reductase inhibition promotes hepatic lipid accumulation in man. J Clin Endocrinol Metab. 2016;101:103‐113. 26574953 10.1210/jc.2015-2928 PMC4701851

[23] Upreti R , Hughes KA , Livingstone DE , et al. 5alpha‐reductase type 1 modulates insulin sensitivity in men. J Clin Endocrinol Metab. 2014;99:E1397‐E1406. 24823464 10.1210/jc.2014-1395 PMC4207930

[24] Ergang P , Vodicka M , Vagnerova K , et al. Inflammation regulates 11beta‐hydroxysteroid dehydrogenase type 1 differentially in specific compartments of the gut mucosal immune system. Steroids. 2017;126:66‐73. 28754259 10.1016/j.steroids.2017.07.007

[25] Abul‐Husn NS , Cheng X , Li AH , et al. A protein‐truncating HSD17B13 variant and protection from chronic liver disease. N Engl J Med. 2018;378:1096‐1106. 29562163 10.1056/NEJMoa1712191 PMC6668033

[26] Ma Y , Belyaeva OV , Brown PM , et al. 17‐beta hydroxysteroid dehydrogenase 13 is a hepatic retinol dehydrogenase associated with histological features of nonalcoholic fatty liver disease. Hepatology. 2019;69:1504‐1519. 30415504 10.1002/hep.30350 PMC6438737

[27] Stewart PM , Burra P , Shackleton CH , Sheppard MC , Elias E . 11 beta‐Hydroxysteroid dehydrogenase deficiency and glucocorticoid status in patients with alcoholic and non‐alcoholic chronic liver disease. J Clin Endocrinol Metab. 1993;76:748‐751. 8445034 10.1210/jcem.76.3.8445034

[28] Arlt W , Biehl M , Taylor AE , et al. Urine steroid metabolomics as a biomarker tool for detecting malignancy in adrenal tumors. J Clin Endocrinol Metab. 2011;96:3775‐3784. 21917861 10.1210/jc.2011-1565 PMC3232629

[29] Estes C , Anstee QM , Arias‐Loste MT , et al. Modeling NAFLD disease burden in China, France, Germany, Italy, Japan, Spain, United Kingdom, and United States for the period 2016–2030. J Hepatol. 2018;69:896‐904. 29886156 10.1016/j.jhep.2018.05.036

[30] McPherson S , Stewart SF , Henderson E , Burt AD , Day CP . Simple non‐invasive fibrosis scoring systems can reliably exclude advanced fibrosis in patients with non‐alcoholic fatty liver disease. Gut. 2010;59:1265‐1269. 20801772 10.1136/gut.2010.216077

