# Supplementary material for: Accurate non‐invasive diagnosis and staging of non‐alcoholic fatty liver disease using the urinary steroid metabolome
Source: Aliment Pharmacol Ther. 2020 Apr 16;51(11):1188–97. doi: 10.1111/apt.15710 (PMC8150165; doi:10.1111/apt.15710)
Supplement: Supplementary file 1 — Table S1‐S4‐Fig S1‐S3 [file APT-51-1188-s001.docx]

**Supplementary appendix:**

***GMLVQ analysis***

GMLVQ analysis of GC-MS data was performed on data from urine sample using a panel of 32 steroids. Subject records with missing steroid measurements were removed prior to analysis. In order to compare the performance of GMLVQ on urinary steroid data against established non-invasive scores of NAFLD stage, analysis in a discrete sub-group where complete data sets were available was performed. Records with missing BMI, diabetes status, ALT, AST, albumin or platelets in the F0-F4 groups were removed; 128 patients remained in this subgroup analysis (control=56, F0-F2=19, F3-F4=53).

Steroid data was log transformed (Log10) before undergoing standardisation by z-score transform during the GMLVQ analysis. Remaining missing values for age and BMI in the control group were treated along the lines of the NaN-LVQ prescription, ignoring them in the computation of the corresponding distances.

All models were checked for gender bias by computing ROC AUC performance for the male and female groups individually. Gender was also considered as a predictor. However, this did not affect the performance of the GMLVQ model and gender was assigned near-zero weight in the models.

Feature selection was used to refine the model to investigate the performance of a reduced number of steroids. The top 10 most relevant steroids were identified from the relevance matrix to reduce the steroid number from 32 to 10. Following this, a backwards elimination ‘greedy search’ strategy was employed to sequentially reduce the number of steroids from 10 to 2 which involved re-training the GMLVQ system each time the least relevant steroid was removed.

Due to the number of subjects in the cohort, repeated random sub-sampling validation was applied to divide the dataset into training and validation sets in order to evaluate GMLVQ performance. The process was repeated to produce 200 results, each one corresponding to a division of 90% for training and 10% for validation. The randomized sets were stratified in the sense that both training and validation sets contained at least one example from each class.

Receiver operating characteristics (ROC) ^15^ and area under curve (AUC) of the ROC curve was used as the primary performance metric to compare newly generated models and various alternative established non-invasive scores for liver fibrosis. Bootstrapping was used to calculate 95% confidence intervals for the mean ROC values and mean feature relevances. 10,000 bootstrap samples were taken from the 200 validation results. Mean values per sample were calculated and the borders of the centre 95% values were used to provide the confidence interval.

***Supplementary Table 1:*** Numerical values assigned to individual steroid metabolites

| No. | Common name | Chemical name |
| --- | --- | --- |
| 1 | Androsterone | 5α-androstan-3α-ol-17-one |
| 2 | Etiocholanolone | 5β-androstan-3α-ol-17-one |
| 3 | 11β-hydroxyandrosterone | 5α-androstane-3α, 11β-diol-17-one |
| 4 | Dehydroepiandrosterone | 5-androsten-3β-ol-17-one |
| 5 | 16α-hydroxy-dehydroepiandrosterone | 5-androstene-3β,16α-diol-17-one |
| 6 | Pregnenetriol | 5-pregnene-3β,17, 20α-triol |
| 7 | Pregnenediol | 5-pregnene-3β, 20α-diol *and* 5, 17, (20)-  pregnadien-3β-ol |
| 8 | Tetrahydro-11-  dehydrocorticosterone | 5β-pregnane-3α, 21-diol, 11, 20-dione |
| 9 | 5α-tetrahydro-11-  dehydrocorticosterone | 5α-pregnane-3α, 21-diol-11, 20-dione |
| 10 | Tetrahydrocorticosterone | 5β-pregnane-3α,11β,21-triol-20-one |
| 11 | 5α-tetrahydrocorticosterone | 5α-pregnane-3α, 11β,21-triol-20-one |
| 12 | 18-hydroxytetrahydro-11-  dehydrocorticosterone | 5β-pregnane-3α, 3,18,21-trihydroxy-11,20-dione |
| 13 | Tetrahydro-11-deoxycorticosterone | 5β-pregnane-3α, 21-diol-20-one |
| 14 | Tetrahydroaldosterone | 5β-pregnane-3α, 11β,21-triol-20-one-18-al |
| 15 | Pregnanediol | 5β-pregnane-3α, 20α-diol |
| 16 | 3α,5α-17-hydroxypregnanolone | 5α-pregnane-3α, 17α-diol-20-one |
| 17 | 17-hydroxypregnanolone | 5β-pregnane-3α, 17α-diol-20-one |
| 18 | Pregnanetriol | 5β-pregnane-3α, 17α,20α-triol |
| 19 | Pregnanetriolone | 5β-pregnane-3α, 17,20α-triol-11-one |
| 20 | Tetrahydro-11-deoxycortisol | 5β-pregnane-3α, 17, 21-triol-20-one |
| 21 | Cortisol | 4-pregnene-11β, 17, 21-triol-3, 20-dione |
| 22 | 6β-hydroxy-cortisol | 4-pregnene-6β, 11β, 17, 21-tetrol-3, 20-dione |
| 23 | Tetrahydrocortisol | 5β-pregnane-3α, 11β,17, 21-tetrol-20 one |
| 24 | 5α-tetrahydrocortisol | 5α-pregnane-3α, 11β,17, 21-tetrol-20-one |
| 25 | α-cortol | 5β-pregnan-3α, 11β, 17,20α, 21-pentol |
| 26 | β-cortol | 5β-pregnan-3α, 11β, 17, 20β, 21-pentol |
| 27 | 11β-hydroxyetiocholanolone | 5β-androstane-3α, 11β-diol-17-one |
| 28 | Cortisone | 4-pregnene-17α, 21-diol-3, 11, 20-trione |
| 29 | Tetrahydrocortisone | 5β-pregnene-3α, 17, 21-triol-11, 20-dione |
| 30 | α-cortolone | 5β-pregnane-3α, 17,20α, 21-tetrol-11 one |
| 31 | β-cortolone | 5β-pregnane-3α, 17,20β, 21-tetrol-11-one |
| 32 | 11-oxoetiocholanolone | 5β-androstan-3α-ol-11,17-dione |

**Supplementary Table 2:** Urinary steroid metabolite analysis

GC/MS analysis of spot urine samples from 106 control and 121 individuals with NAFLD stratified by fibrosis stage. (THE = tetrahydrocortisone, THF = tetrahydrocortisol, UFF = urinary free cortisol, UFE = urinary free cortisone, 11OH-androst = 11hydroxyandrosterone, 11OH-etio = 11hydroxyetiocholanolone, 11oxo-etio = 11oxo-etiocholanolone, Total glucocorticoid metabolites = cortisol+6β-OH-Cortisol+THF+5αTHF+α-cortol+β-cortol+11b-OH-ETIO+ cortisone+THE+α-cortolone+β-cortolone+11-oxo-etio, Fm = cortisol+THF+5αTHF+α-cortol+β-cortol, Em = cortisone+THE+α-cortolone+β-cortolone). Statistical analysis was performed on log transformed steroid values or ratios, * p<0.05 *vs.* control; § p<0.05 *vs.* F0-2.

|  | Control | NAFLD  F0-2 | NAFLD  F3-4 |
| --- | --- | --- | --- |
| ***Urine steroid metabolites, μg/g urinary creatinine (mean* ±*SEM****)* | | | |
| Androsterone (An) | 959 ± 53 | 1787 ± 328 | 835 ± 81*^§^ |
| Etiocholanolone (Et) | 918 ± 56 | 1029 ± 121 | 466 ± 55*^§^ |
| 11β-hydroxyandrosterone (11OH-An) | 442 ± 21 | 630 ± 87 | 642 ± 46* |
| Dehydroepiandrosterone (DHEA) | 249 ± 44 | 428 ± 103 | 139 ± 39*^§^ |
| 16α-hydroxy-dehydroepiandrosterone | 287 ± 31 | 347 ± 54 | 387 ± 49 |
| Pregnenetriol (5-PT) | 156 ± 13 | 284 ± 37* | 165 ± 20^§^ |
| Pregnenediol (5-PD) |  |  |  |
| Tetrahydro-11-  dehydrocorticosterone (THA) | 97 ± 7 | 101 ± 9 | 83 ± 9*^§^ |
| 5α-tetrahydro-11-  Dehydrocorticosterone (5αTHA) | 87 ± 3 | 80 ± 7 | 60 ± 7*^§^ |
| Tetrahydrocorticosterone (THB) | 101 ± 8 | 107 ± 11 | 98 ± 14^§^ |
| 5α-tetrahydrocorticosterone (5αTHB) | 219 ± 14 | 296 ± 31 | 217 ± 24^§^ |
| 18-hydroxytetrahydro-11-  dehydrocorticosterone (18OH-THA) | 44 ± 3. | 46 ± 4 | 57 ± 5 |
| Tetrahydro-11 deoxycorticosterone (TH-DOC) | 14 ± 1 | 12 ± 1 | 9 ±1*^§^ |
| Tetrahydroaldosterone (3α5βTHaldo) | 30 ± 2 | 26 ± 2 | 43.7 ± 4*^§^ |
| Pregnanediol (PD) | 161 ± 16 | 132 ±17 | 128 ± 29* |
| 3α,5α-17-hydroxypregnanolone (3α5α17HP) | 9 ± 1 | 15 ± 2 | 12 ± 1 |
| 17-hydroxypregnanolone (17HP) | 85 ± 7 | 89 ± 9 | 79 ± 11^§^ |
| Pregnanetriol (PT) | 323 ± 16 | 316 ± 24 | 234 ± 19*^§^ |
| Pregnanetriolone (PTONE) | 25 ± 6 | 16 ± 2 | 15 ± 2^§^ |
| Tetrahydro-11-deoxycortisol (THS) | 68 ± 5 | 65 ± 7 | 80 ±10 |
| Cortisol (F) | 56 ± 6 | 87 ± 14* | 139 ± 16*^§^ |
| 6β-hydroxy-cortisol (6βOHF) | 94 ± 5 | 122 ± 19 | 161 ± 16* |
| Tetrahydrocortisol (THF) | 1389 ± 69 | 1583 ±130 | 1512 ± 145 |
| 5α-tetrahydrocortisol (5αTHF) | 1114 ± 54 | 1669 ± 211 | 1682 ±126* |
| α-cortol | 269 ±14 | 334 ± 23* | 406 ± 35* |
| β-cortol | 380 ± 20 | 381 ± 25 | 434 ± 28 |
| 11β-hydroxyetiocholanolone (11OHEt) | 228 ± 16 | 122±16* | 123 ± 13* |
| Cortisone (E) | 78 ± 4 | 104 ± 14 | 191 ± 17*^§^ |
| Tetrahydrocortisone (THE) | 2835 ± 130 | 3021 ± 223 | 2607 ± 225^§^ |
| α-cortolone | 1130 ± 52 | 1297 ± 79 | 1282 ± 86 |
| β-cortolone | 551 ± 24 | 526 ± 30 | 620±44 |
| 11-oxoetiocholanolone (11oxoEt) | 296 ± 19 | 168 ± 16* | 123 ± 12* |
| Total glucocorticoid metabolites (GCm) | 8072 ± 306 | 9415 ± 656 | 9282 ± 638 |
| Total cortisol metabolites (Fm) | 3208 ± 133 | 4054 ± 351 | 4174 ± 308 |
| Total cortisone metabolites (Em) | 4595 ± 192 | 4949 ± 311 | 4701 ± 336 |
| ***Corticosteroid metabolite ratios (mean* ±*SEM****)* | |  | |
| *11β-HSD2 activity* | | | |
| UFF/UFE (cortisol/cortisone) | 0.7 ± 0 | 0.8 ± 0.1* | 0.7 ± 0^§^ |
| *11β-HSD1 activity* | | | |
| (THF+5αTHF)/THE | 0.9 ± 0 | 1.1 ± 0.1 | 1.5 ± 0.1* |
| Cortols/cortolones ( (α-cortol+ β-cortol)/( α-cortolone+ β-cortolone) ) | 0.4 ± 0 | 0.4 ± 0 | 0.5 ± 0*^§^ |
| *A-ring reductase activity* | | | |
| 5αTHF/THF | 0.9 ± 0 | 1.1 ± 0.1 | 1.4 ± 0.1*^§^ |
| Androsterone/Etiocholanolone | 1.2 ± 0.1 | 1.8 ± 0.2* | 2.5 ± 0.2* |

***Supplementary Table 3:*** Demographic details of 108 subjects with cirrhosis (F4): 60 with NAFLD cirrhosis and 48 with cirrhosis due to excess alcohol consumption. Data are expressed are mean ± standard deviation (unless otherwise stated) (* p<0.05).

|  | NAFLD cirrhosis | Alcohol cirrhosis | p-value |
| --- | --- | --- | --- |
| N (m/f) (males, %) | 60 (26 / 34) (43) | 48 (33 /15) (69) | 0.29 |
| Age, years | 65 ± 9 | 58 ± 11 | <0.01 |
| BMI, kg/m^2^ | 33.2 ± 5.9 | 28.2 ± 5.9 | <0.01 |
| Proportion with Type 2 Diabetes, % | 70 | 26 | <0.01 |
| Platelets, 10^9^/L | 173 ± 67 | 146 ± 43 | 0.21 |
| ALT, IU/L | 39 ± 19 | 33 ± 25 | 0.08 |
| AST, IU/L | 41 ± 17 | 52 ± 56 | 0.32 |
| Fib-4 Score | 2.9 ± 1.8 | 3.2 ± 1.6 | 0.32 |
| NAFLD Fibrosis Score | 0.90 ± 1.6 | 0.70 ± 1.39 | 0.7 |

***Supplementary table 4*:** Comparison of GMLVQ and GMLVQ* analysis of urinary steroid metabolites *vs.* serum assessments using FIB-4 and NAFLD fibrosis scores using urinary steroid metabolites uncorrected for urinary creatinine and quantified as μg/1000mL urine.

|  | **AUC ROC (95% confidence intervals)** | | | | | |
| --- | --- | --- | --- | --- | --- | --- |
| **Clinical comparison**  **(NAFLD stage)** | **NAFLD Fibrosis score** | **FIB-4** | **GMLVQ**  **(32 steroids)** | **GMLVQ***  **(32 steroids, age, BMI)** | **GMLVQ-10**  **(top 10 steroid metabolites)** | **GMLVQ-10***  **(top 10 steroid metabolites, age, BMI)** |
| *F0-F2 vs. F3-F4* | 0.87 (0.86-0.88) | 0.91 (0.89-0.92) | 0.87 (0.85-0.89) | 0.92 (0.91-0.94) | 0.87 (0.86-0.89) | 0.92 (0.90-0.94) |
| *F0-F3 vs. F4* | 0.87 (0.86-0.88) | 0.84 (0.83-0.85) | 0.85 (0.83-0.87) | 0.90 (0.89-0.91) | 0.85 (0.83-0.87) | 0.89 (0.87-0.91) |
| *Controls vs. F0-F4* |  | | 0.94 (0.93-0.95) | 0.94 (0.93-0.95) | 0.96 (0.95-0.97) | 0.96 (0.95-0.96) |
| *Controls vs. F3-F4* |  |  | 0.99 (0.99-1.00) | 0.99 (0.98-0.99) | 1.00 (0.99-1.00) | 0.99 (0.99-1.00) |
| *Controls vs. F4* |  |  | 1.00 (1.00-1.00) | 1.00 (1.00-1.00) | 1.00 (1.00-1.00) | 1.00 (1.00-1.00) |

***Supplementary Figure. 1.* GMLVQ analysis of patients with NAFLD compared to controls.** Numerical values are given for each individual steroid metabolite (Supplementary Table 1). Two-dimensional visualization of steroid data obtained by projection of the z-score transformed and log-scaled excretion values onto the first and second eigenvector of the relevance matrix (a). Prototypical representatives of disease classes (controls and NAFLD fibrosis stages) using z-score transformed log-scaled steroid excretion values. Diagonal elements of the relevance matrix (normalized to sum 1), indicating the importance of individual steroids in the GMLVQ classifier (c).

***Supplementary Figure 2:*** GMLVQ’ analysis permits very good separation between NAFLD cirrhosis and alcohol related cirrhosis (a). ROC AUC analysis demonstrates potential clinical utility in determining underlying cirrhosis aetiology (b).**
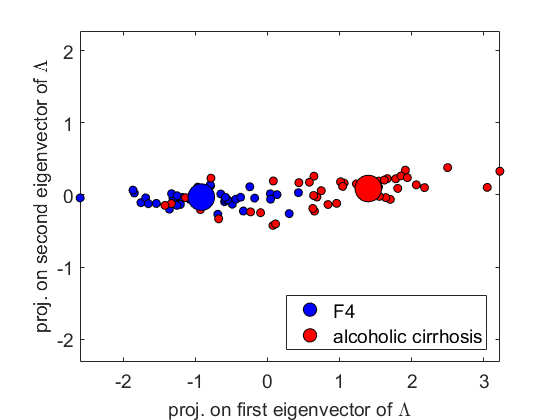

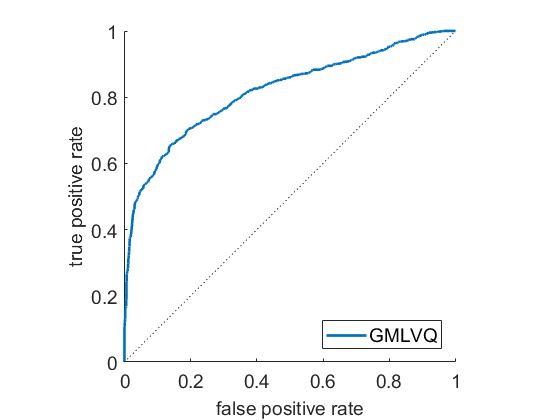
**

ROC AUC

GMLVQ’: 0.83 (0.81 – 0.85)

a

b

***Supplementary Figure 3***: In patients with established NAFLD, GMLVQ analysis is unable to distinguish between patients with NASH (NAFLD Activity Score, NAS>4) and those without NASH (NAS<=4).
